# EFFICACY OBSERVATION OF COMBINED TRANSCUTANEOUS VAGUS NERVE STIMULATION AND TRANSCRANIAL DIRECT CURRENT STIMULATION ON GAIT IN 169 SUBACUTE STROKE PATIENTS

**DOI:** 10.2340/jrm.v56.40348

**Published:** 2024-11-07

**Authors:** Litong WANG, Likai WANG, Zhan WANG, Hongyu ZHAO, Jingyi WU, Fei GAO, Hong TANG

**Affiliations:** 1 School of Biomedical Engineering, Faculty of Medicine, Dalian University of Technology, Dalian; 2 Rehabilitation Medicine Department, The Second Hospital of Dalian Medical University, Dalian; 3 Lab of Intelligent System, School of Control and Engineering, Faculty of Electronic Information and Electrical Engineering, Dalian University of Technology, Dalian, China

**Keywords:** stroke, motor impairment, taVNS, tDCS, gait analysis

## Abstract

**Objective:**

To investigate the combined effect of transcranial magnetic stimulation (TMS) and transcranial direct current stimulation on improving lower limb function in stroke patients.

**Design:**

Randomized controlled trial.

**Subjects/Patients:**

Subacute stroke patients.

**Methods:**

169 post-stroke hemiplegia patients were randomly divided into 4 groups (control, transcranial direct current stimulation, transcutaneous auricular vagus nerve stimulation, and transcutaneous auricular vagus nerve stimulation combined with transcranial direct current stimulation) and evaluated using the Fugl-Meyer Assessment-Lower Extremity (FMA-LL), Timed Up-and-Go (TUG) test, Modified Barthel Index (MBI), Berg Balance Scale (BBS), gait parameters, and surface electromyography (sEMG).

**Results:**

Significant improvements in FMA-LL, MBI, BBS, TUG, gait parameters, and sEMG were noted in the intervention groups compared with the control, with the transcutaneous auricular vagus nerve stimulation combined with transcranial direct current stimulation group showing the most pronounced improvements. Differences in some outcomes were also notable between the transcutaneous auricular vagus nerve stimulation and transcranial direct current stimulation groups.

**Conclusion:**

The combination of transcutaneous auricular vagus nerve stimulation and transcranial direct current stimulation effectively enhances gait, balance, and daily living activities in subacute stroke patients. These benefits are likely due to transcutaneous auricular vagus nerve stimulation activating the solitary and trigeminal nuclei and transcranial direct current stimulation stimulating the motor cortex. Wearable gait analysis systems and electromyography are valuable in clinical gait assessment for these patients.

## LAY ABSTRACT

Transcutaneous auricular vagus nerve stimulation and transcranial direct current stimulation are non-invasive neuromodulation techniques with potential in stroke rehabilitation. This study investigates their combined effect on enhancing lower limb function in stroke patients, targeting both peripheral and central nervous system pathways to improve motor recovery and neurorehabilitation strategies. Transcutaneous auricular vagus nerve stimulation and transcranial direct current stimulation (combined or alone) combined with physical therapy were used in the 3 groups and compared with the control group. Compared with the control group, the lower limb function and gait performance of the 3 groups were significantly improved. This suggests that combination therapy may be a valuable addition to rehabilitation programmes for lower limb function in post-stroke patients.

Stroke often leads to persistent motor impairments, with gait dysfunction being one of the most common and debilitating outcomes, significantly hindering recovery (1, 2). Despite advancements in rehabilitative techniques, a significant proportion of stroke survivors continue to experience substantial mobility limitations, impacting their quality of life and increasing the risk of secondary health complications (3). Traditional rehabilitation methods are only partially effective, necessitating the exploration of novel therapeutic strategies to enhance neural plasticity and functional recovery during the critical subacute phase, which is known for its high potential for neural adaptations (4, 5).

Transcranial direct current stimulation (tDCS) has been extensively studied over the past 2 decades, with numerous studies demonstrating its potential to modulate cortical excitability and promote motor recovery in stroke patients. For example, randomized controlled trials have shown that anodal tDCS over the motor cortex can enhance motor learning by increasing cortical excitability and synaptic plasticity, leading to improvements in motor function (6–8). However, the effectiveness of tDCS is influenced by several factors, including the intensity and duration of stimulation, the timing of intervention relative to motor training, and individual patient characteristics (9, 10). Despite promising results, there remain challenges in optimizing stimulation protocols to achieve consistent and clinically meaningful outcomes across diverse patient populations (11). Additionally, the spatial precision of tDCS remains a concern, as its effects are not limited to the targeted area, leading to potential off-target effects that may complicate interpretation and application (10).

Transcutaneous auricular vagus nerve stimulation (taVNS) is a more recent neuromodulation technique that has garnered attention for its ability to non-invasively stimulate the vagus nerve, thereby influencing autonomic and central nervous system functions (12). Research has demonstrated that taVNS can activate brainstem nuclei and modulate cortical networks involved in motor control and neuroplasticity (13). Preclinical and early clinical studies have indicated potential benefits in enhancing motor recovery and reducing post-stroke complications such as spasticity and pain (14, 15). However, the exact mechanisms through which taVNS exerts its effects on motor recovery are still not fully understood, and further research is needed to elucidate these pathways and determine the most effective stimulation parameters. Additionally, while taVNS is generally well tolerated, optimizing its application for different patient groups remains an area of ongoing investigation (14, 16).

Given the complementary mechanisms of action of tDCS and taVNS – where tDCS primarily modulates cortical excitability and taVNS broadly activates neural circuits – there is a growing interest in exploring the combined use of these techniques to enhance motor recovery post-stroke. The potential for synergy between these modalities lies in their ability to target different aspects of neural plasticity: tDCS may prime the motor cortex for enhanced responsiveness to subsequent stimuli, while taVNS could create a neurophysiological environment conducive to widespread plastic changes (17). However, despite preliminary evidence suggesting that such synergy exists, particularly in cognitive domains (18), research specifically addressing the combined effects on motor function is limited. This study aims to address this gap by systematically investigating whether the concurrent application of tDCS and taVNS can lead to superior improvements in gait and lower limb function compared with each modality alone.

## MATERIALS AND METHODS

### Patients

**Fig. 1:**
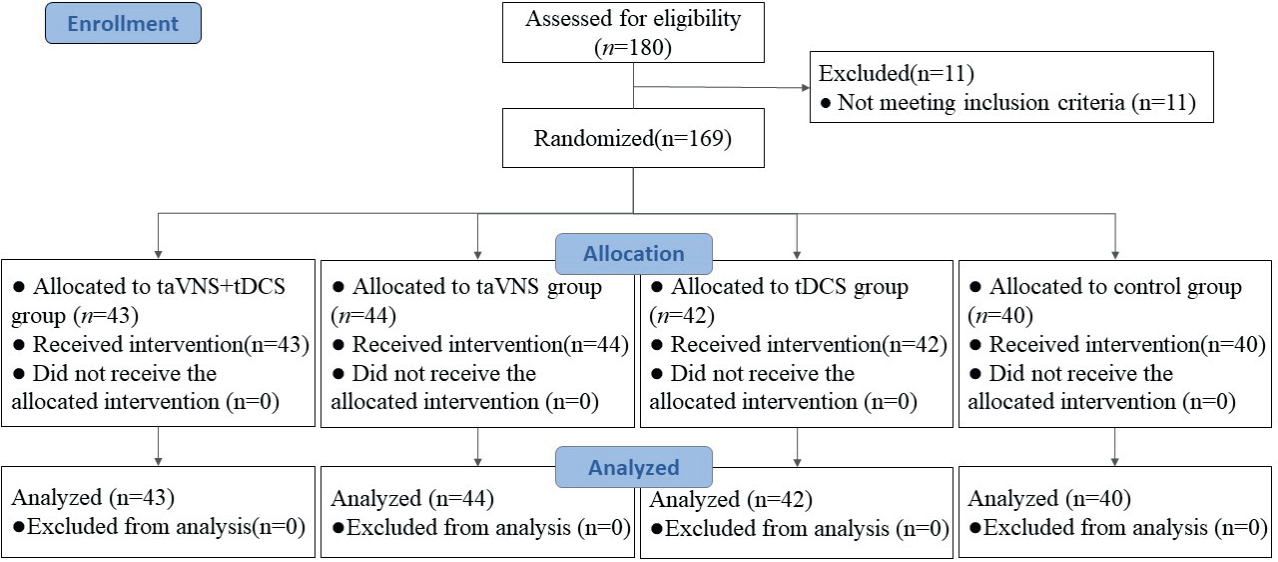
Consolidated Standards of Reporting Trials (CONSORT) flow diagram of patients enrolled in the trial for randomization.

Participants for this study were enrolled from the outpatient and inpatient rehabilitation units of the Second Affiliated Hospital of Dalian Medical University, spanning a period from June 2019 to June 2022. Institutional ethical clearance was obtained from the hospital’s ethics committee prior to the commencement of the study. The patient cohort was systematically allocated into 4 distinct groups through a random number table methodology. These groups included a control group, a group receiving tDCS, a group undergoing taVNS, and a combined therapy group receiving taVNS with tDCS. Each group comprised 45 patients, totalling 180 participants. Of these, 169 patients successfully completed the study. Fig. 1 shows the Consolidated Standards of Reporting Trials (CONSORT) flow diagram, which illustrates the enrolment and randomization of patients in the trial. Notably, statistical analysis revealed no significant disparities in terms of gender, age, disease duration, stroke type, or paralysis side across the 4 groups (*p* > 0.05), as delineated in Table I. This homogeneity in baseline characteristics ensured a balanced comparison across the treatment and control cohorts. All patients provided signed informed consent and were informed of potential adverse events prior to the trial. This study protocol was approved by the Ethics Committee of the Second Affiliated Hospital of Dalian Medical University (approval number: 2023-058). The trial was registered with the China Clinical Trial Registration Center (www.chictr.org.cn, registration number: ChiCTR2300069403). All participants provided informed consent prior to the start of the trial, and they were free to leave at any time. The Declaration of Helsinki and all pertinent rules and regulations were followed during every procedure.

**Table I:** Comparison of general data among the 4 groups

Group	taVNS+tDCS group (*n* = 43)	taVNS group (*n* = 44)	tDCS group (*n* = 42)	Control group (*n* = 40)
Gender, *n* (%)				
Male	22 (51.2)	21 (47.7)	18 (42.8)	17 (42.5)
Female	21 (48.8)	23 (52.3)	24 (57.1)	23 (57.5)
Age, years, mean (SD)	61.54 (5.78)	60.82 (6.19)	63.16 (5.75)	61.94 (3.28)
Course of disease, day, mean (SD)	18.98 (4.56)	19.24 (5.83)	20.05 (4.28)	18.89 (5.17)
Type, *n* (%)				
Cerebral infarction	18 (41.9)	24 (54.5)	19 (45.2)	16 (40)
Cerebral haemorrhage	25 (58.1)	20 (45.5)	23 (54.8)	24 (60)
Hemiplegic side, *n* (%)				
Right	23 (53.5)	18 (40.9)	20 (47.6)	21 (52.5)
Left	20 (46.5)	26 (59.1)	22 (52.4)	19 (47.5)

Included and dropout patients did not differ significantly on any of the characteristics (all *p* > 0.05).

SD: standard deviation; taVNS: transcutaneous auricular vagus nerve stimulation; tDCS: transcranial direct current stimulation.

### Diagnostic criteria

The diagnosis of stroke in this study adhered to the criteria outlined in the “Diagnosis of Major Cerebrovascular Diseases in China 2019”. Each participant presented with clinically evident neurological symptoms and signs. Diagnostic confirmation was achieved through neuroimaging techniques, specifically head computed tomography (CT) or magnetic resonance imaging (MRI), which ascertained the presence of either cerebral infarction or cerebral haemorrhage in the patients.

### Inclusion criteria

The study stipulated specific criteria for participant inclusion: (*i*) Confirmed diagnosis of stroke, with the patient in a stable medical condition. (*ii*) First occurrence of stroke characterized by a unilateral lesion, with an onset time ranging from 14 to 45 days. (*iii*) Age bracket of 18 to 70 years, encompassing all genders. (*iv*) A Brunnstrom stage of 3 or higher for lower limb functionality. (*v*) Obtained informed consent, where both the patients and their family members were adequately informed about the study, agreed to participate, and signed the informed consent form.

### Exclusion criteria

The study meticulously outlined a set of exclusion criteria to ensure patient safety and the integrity of the research outcomes: (*i*) Patients with lower limb joint disorders, including those with limited joint mobility due to joint replacement surgery, or back and leg pain impairing walking ability. (*ii*) Individuals exhibiting unstable vital signs, consciousness disorders, or an inability to cooperate with examination and treatment procedures. (*iii*) Patients with cognitive impairments, as indicated by a Mini-Mental State Examination score below 27. (*iv*) Individuals suffering from severe conditions in the cardiovascular, digestive, or endocrine systems, or other significant health issues. (*v*) Patients contraindicated for ear stimulation due to conditions like infections, ulcers, or scars on the earlobe. (*vi*) Those with a heart rate below 60 beats per minute, or with implantable devices such as pacemakers or cochlear implants. (*vii*) Individuals who have undergone vagus nerve surgery or possess metal objects in their skull. (*viii*) Patients deemed unsuitable for tDCS due to factors like cranial metal implants or hypersensitivity, injury, or inflammation in the stimulation area. (*ix*) Patients presenting with other medical or health conditions contraindicating the proposed treatments.

### Dropout criteria

To maintain the integrity and reliability of the study, specific dropout criteria were established: (*i*) Patients experiencing a deterioration in their baseline medical condition. (*ii*) Individuals who discontinued the treatment due to adverse reactions. (*iii*) Patients who encountered serious adverse reactions necessitating an interruption of the treatment. (*iv*) Participants who received treatments outside of the study protocol. Instances falling into these categories were classified as dropout cases.

### Treatment methods

**Fig. 2:**
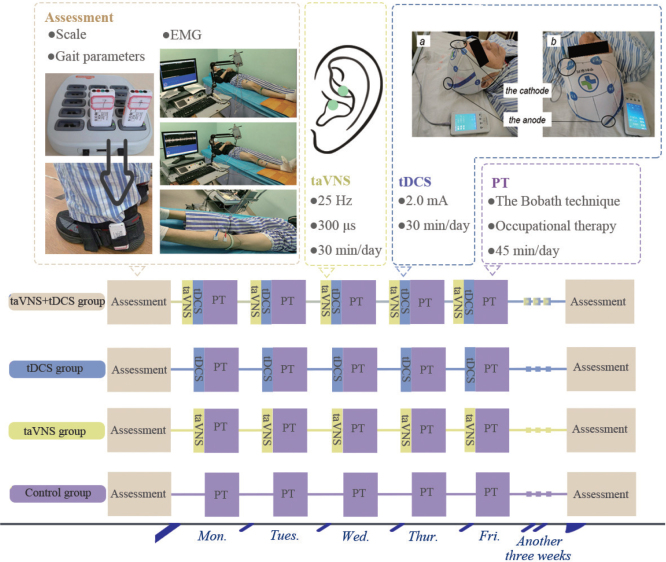
Experimental design diagram. PT: physical therapy, which refers to basic rehabilitation training.

The methodology employed in this study encompassed the administration of secondary stroke prevention medications to all patient groups. These medications included antihypertensives, hypoglycaemic agents, antiplatelet aggregation agents, and lipid-lowering drugs. The approach was standardized across the 4 groups to ensure a consistent therapeutic baseline. Blinding was incorporated into the study design, with both patients and outcome assessors blinded to the group assignments to minimize bias. For the control group, the rehabilitation regimen was grounded in the principles of the Bobath technique and supplemented with occupational therapy. Each patient in this group underwent 1 training session daily, each lasting 45 min, conducted 5 times a week over a 4-week period. In addition to this, all patients continued to receive standard rehabilitation care as provided by the stroke unit, including physiotherapy and other supportive therapies, which were not part of the study protocol but were ethically necessary to ensure comprehensive care during the sub-acute phase. The tDCS group received additional tDCS prior to their standard rehabilitation training, while the taVNS group were treated with taVNS before their rehabilitation exercises. All stimulation interventions in these groups were administered prior to the rehabilitation sessions, not simultaneously, to explore the potential priming effects of these neuromodulation techniques on subsequent physical rehabilitation. The fourth group, receiving a combination of therapies, underwent simultaneous taVNS and tDCS treatment prior to their rehabilitation sessions, as depicted in Fig. 2. This integrative approach was designed to assess the potential synergistic effects of combining these neuromodulation techniques with conventional rehabilitation.

*Transcranial direct current stimulation treatment.* In the implementation of the tDCS treatment, the MBM-IV400 device (Jiangxi Huaheng Jingxing Medical Technology Co, Jiangxi, China) was utilized. The placement of electrodes was strategically determined: the anode was positioned over the M1 cortex area corresponding to the lesioned hemisphere of the brain, while the cathode was situated on the supraorbital area of the opposite side (see Fig. 2 for detailed placement). The treatment protocol involved delivering a current of 2.0 mA. Each session lasted for 30 min, administered daily, 5 times a week. This regimen was maintained consistently over a 4-week period. This protocol was devised to maximize the therapeutic benefits of tDCS while ensuring patient comfort and safety.

tDCS is generally safe for research and clinical use, though it carries some risks. Common mild side effects include itching, tingling, headaches, and discomfort (19). Serious events, such as seizures or skin lesions, are rare, with no direct link to tDCS established. Extensive trials involving over 33,200 sessions have shown no documented serious adverse events, highlighting tDCS’s favourable safety profile (20). To minimize risks, it is crucial to conduct thorough pre-screening for contraindications, monitor participants during sessions, carefully adjust stimulation parameters, and adhere to safety guidelines established by organizations like the International Federation of Clinical Neurophysiology (19).

*Transcutaneous auricular vagus nerve stimulation treatment.* For the taVNS treatment, the study utilized the En-stim4 device, provided by Shanghai Xibei Electronics Technology Development Co., Shanghai, China. This device features 2 electrodes, each being a 5×5mm circular metal electrode, which were specifically placed on the tragus of the left ear. This location is recognized as the distribution area of the vagus nerve, as depicted in Fig. 2. The parameters for the stimulation were precisely set: the frequency was established at 25 Hz, and the pulse width was 300 μs. The stimulation protocol involved a 30-s stimulation period followed by a 30-s interval, employing biphasic sinusoidal pulses. The current intensity was incrementally increased from the lowest level until the patient experienced discomfort. The final current setting was then finely adjusted to be just below the threshold of pain sensation. The treatment schedule mirrored that of the tDCS protocol, with sessions conducted once daily, each lasting 30 min, 5 days a week, over a 4-week duration. This consistent and carefully monitored approach was designed to optimize the therapeutic effect of taVNS while ensuring patient comfort and safety.

taVNS is generally safe but comes with some risks. Common side effects include ear pain, headache, tingling, and skin irritation at the stimulation site. A review of 177 studies found a low incidence of adverse events, with no significant difference between active and control groups (21). Serious events like tinnitus and facial droop are rare, and no causal link to taVNS has been established (22). To minimize risks, it is crucial to screen participants, monitor sessions, adjust stimulation parameters carefully, and document any adverse events.

### Efficacy evaluation

To ensure consistency and reliability in the evaluation of treatment outcomes, the same evaluator was responsible for conducting all assessments. These evaluations included scale assessments, gait detection, and surface electromyography measurements of the lower limbs, both before the initiation of treatment and 4 weeks post-treatment.

### Observation indicators

The observation indicators for this study comprised the Fugl-Meyer Assessment of Lower Extremity (FMA-LL), Timed-Up-and-Go (TUG) test, Modified Barthel Index (MBI), and Berg Balance Scale (BBS) to assess lower limb motor function in patients across 4 groups before and after 4 weeks of treatment. Gait parameters such as step cycle, swing phase time, stride length, ankle dorsiflexion, step height, and walking speed were collected. Additionally, mean and integrated electromyography (EMG) values for specific leg muscles were measured.

For lower limb motor function, the FMA-LL’s Lower Limb section, containing 17 items with a total score of 34, was used. The TUG test involved patients standing from a chair, walking, and returning, timed with a stopwatch. Gait analysis was performed using the Shimmer Company’s gait analysis system (Shimmer Research, Dublin Ireland), with the strap placement detailed in Fig. 2. Surface EMG measurements employed the Viking Quest device, Madison, USA, recording signals from specific muscles (as shown in Fig. 2). Skin preparation and electrode placement followed specific protocols, and EMG data were analysed using MyoResearch software (https://www.noraxon.com/our-products/myoresearch), focusing on mean and integrated EMG values. The MBI scale evaluated patients’ self-care abilities in daily life activities, with a total score of 100 indicating various levels of functional impairment or ability. Balance function was assessed using the BBS, encompassing 14 items for static and dynamic balance, with a maximum score of 56. Scores below 40 suggested an increased risk of falls.

### Statistical processing

SPSS 25.0 statistical software was used for analysis (IBM Corp, Armonk, NY, USA). The data of patients in the same group before and after rehabilitation treatment were analysed by paired sample *t*-test, the comparison between the same parameter groups of patients in the 4 groups was analysed by one-way ANOVA, and the post hoc comparison was analysed by LSD test. *P* < 0.05 after analysis indicated statistical difference.

## RESULTS

### Comparative analysis of FMA-LL, MBI, BBS, and TUG scores before and after treatment across the 4 groups

**Fig. 3:**
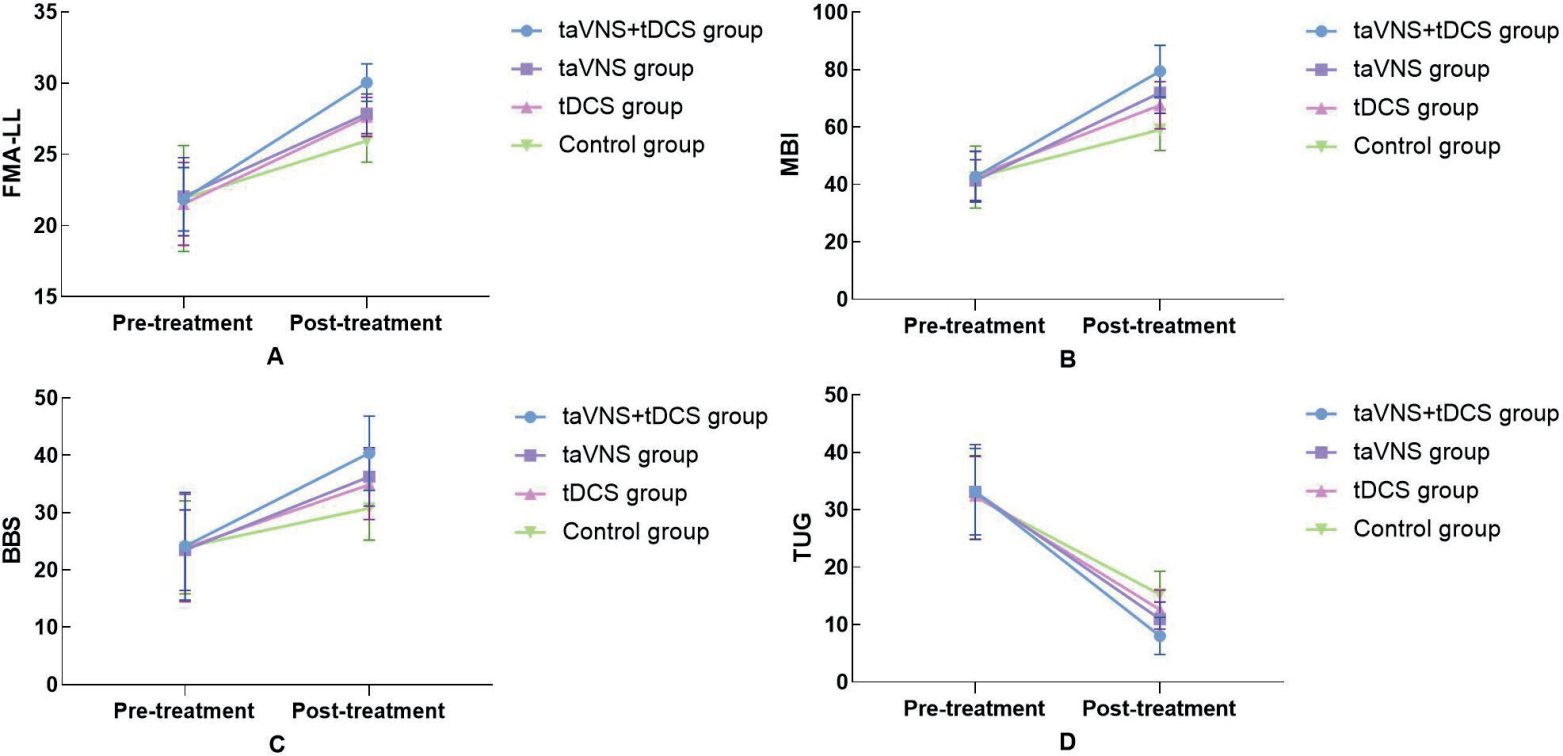
Comparison of scale scores in 4 groups before and after treatment.

The study conducted a comprehensive comparative analysis of the FMA-LL, MBI, BBS, and TUG scores across 4 groups, before and after treatment. Initially, no statistically significant differences were observed among the groups in baseline scores (*p* > 0.05), ensuring a uniform starting point for evaluating treatment effects. Post-treatment, the taVNS combined with tDCS group exhibited significant improvements across all metrics: FMA-LL scores rose from 21.85 (2.23) to 30.04 (1.32), MBI scores increased from 42.67 (8.78) to 79.45 (9.03), BBS scores improved from 24.14 (9.36) to 40.38 (6.47), and TUG scores decreased from 33.16 (7.51) s to 8.03 (3.23) s, all indicating substantial enhancements in motor function, daily living activities, balance, and mobility (*p* < 0.05), as detailed in Fig. 3. These improvements were notably more pronounced compared with the taVNS and tDCS groups, which also showed enhancements but to a lesser extent. This comparative effectiveness, underscored by significant statistical differences as evidenced by the F values from the ANOVA, and detailed in Table II, highlights the superior efficacy of the combined taVNS and tDCS therapy over single-modality treatments or control conditions, suggesting a promising avenue for optimizing stroke rehabilitation strategies.

**Table II:** Between-group comparison of scales, gait parameters, and EMG after the intervention (sample mean)

Item	Pre-/post-treatment	taVNS+tDCS group	taVNS group	tDCS group	CG	*F*	*p*-value
FMA-LL	Pre	21.85 ± 2.23	22.04 ± 2.74	21.53 ± 2.90	21.91 ± 3.72	0.245	0.657
	Post	30.04 ± 1.32	27.85 ± 1.39	27.63 ± 1.38	25.95 ± 1.49	21.421	< 0.001***
MBI	Pre	42.67 ± 8.78	41.38 ± 7.25	43.03 ± 8.58	42.57 ± 10.82	0.624	0.478
	Post	79.45 ± 9.03	71.92 ± 7.11	67.60 ± 8.21	59.14 ± 7.23	18.965	< 0.001***
BBS	Pre	24.14 ± 9.36	23.46 ± 7.01	23.85 ± 9.36	23.97 ± 8.10	0.258	0.647
	Post	40.38 ± 6.47	36.26 ± 5.11	34.91 ± 6.09	30.75 ± 5.52	14.569	< 0.001***
TUG	Pre	33.16 ± 7.51	33.09 ± 8.27	32.47 ± 6.82	32.19 ± 7.26	0.401	0.574
	Post	8.03 ± 3.23	10.97 ± 2.95	12.62 ± 3.41	15.28 ± 4.05	16.784	< 0.001***
Gait cycle (s)	Pre	2.06 ± 0.41	2.03 ± 0.70	2.05 ± 0.36	2.04 ± 0.33	0.0460	0.797
	Post	1.07 ± 0.12	1.32 ± 0.14	1.47 ± 0.24	1.58 ± 0.09	15.906	< 0.001***
Swing phase time (s)	Pre	0.63 ± 0.02	0.60 ± 0.09	0.62 ± 0.04	0.61 ± 0.06	0.056	0.684
	Post	0.86 ± 0.06	0.78 ± 0.04	0.75 ± 0.02	0.70 ± 0.06	0.096	< 0.001***
Stride length (cm)	Pre	58.89 ± 4.97	59.27 ± 5.06	60.04 ± 4.54	59.86 ± 5.31	11.182	0.679
	Post	93.57 ± 12.14	83.86 ± 8.92	76.01 ± 6.52	72.87 ± 6.01	13.559	< 0.001***
Ankle dorsiflexion (°)	Pre	6.52 ± 0.99	6.48 ± 1.24	6.36 ± 1.18	6.57 ± 1.64	0.229	0.633
	Post	17.98 ± 1.02	15.03 ± 2.11	13.21 ± 1.94	10.02 ± 1.15	12.478	< 0.001***
Stride height (m)	Pre	0.06 ± 0.06	0.07 ± 0.08	0.06 ± 0.09	0.07 ± 0.05	0.317	0.707
	Post	0.21 ± 0.04	0.18 ± 0.07	0.15 ± 0.07	0.12 ± 0.13	0.621	< 0.001***
Stride speed (cm/s)	Pre	39.54 ± 1.68	40.67 ± 1.94	39.62 ± 1.71	40.04 ± 2.07	15.127	0.834
	Post	73.96 ± 5.34	68.92 ± 6.01	64.12 ± 7.22	59.50 ± 7.08	21.531	< 0.001***
AEMG (mv) Δ	Pre	0.05 ± 0.02	0.06 ± 0.05	0.06 ± 0.04	0.05 ± 0.05	0.232	0.218
	Post	0.15 ± 0.07	0.13 ± 0.06	0.11 ± 0.04	0.08 ± 0.06	0.078	< 0.001***
iEMG (μVs) Δ	Pre	0.06 ± 0.02	0.05 ± 0.01	0.06 ± 0.02	0.05 ± 0.03	0.067	0.478
	Post	0.16 ± 0.03	0.13 ± 0.07	0.10 ± 0.02	0.08 ± 0.03	0.941	< 0.001***
AEMG (mv) θ	Pre	0.06 ± 0.03	0.05 ± 0.02	0.06 ± 0.01	0.05 ± 0.04	0.214	0.687
	Post	0.17 ± 0.06	0.14 ± 0.07	0.11 ± 0.05	0.08 ± 0.02	0.871	< 0.001***
iEMG (μVs) θ	Pre	0.05 ± 0.01	0.06 ± 0.03	0.05 ± 0.03	0.06 ± 0.02	0.324	0.614
	Post	0.15 ± 0.03	0.13 ± 0.02	0.11 ± 0.01	0.08 ± 0.04	0.987	< 0.001***
AEMG (mv) Φ	Pre	0.06 ± 0.01	0.05 ± 0.02	0.06 ± 0.03	0.06 ± 0.04	0.178	0.367
	Post	0.16 ± 0.03	0.14 ± 0.06	0.11 ± 0.01	0.08 ± 0.03	0.378	< 0.001***
iEMG (μVs) Φ	Pre	0.05 ± 0.05	0.06 ± 0.04	0.06 ± 0.01	0.06 ± 0.02	0.049	0.701
	Post	0.17 ± 0.03	0.14 ± 0.02	0.11 ± 0.01	0.09 ± 0.04	0.164	< 0.001***

taVNS: transcutaneous auricular vagus nerve stimulation; tDCS: transcranial direct current stimulation; CG: control group. Δ: quadriceps muscle; θ: anterior tibial muscle; Φ: the lateral head muscle.

*p < 0.05, **p < 0.01,

*** *p* < 0.001, where *p* < 0.05 suggests statistical significance.

### Comparative analysis of gait parameters before and after treatment among the 4 groups

**Fig. 4:**
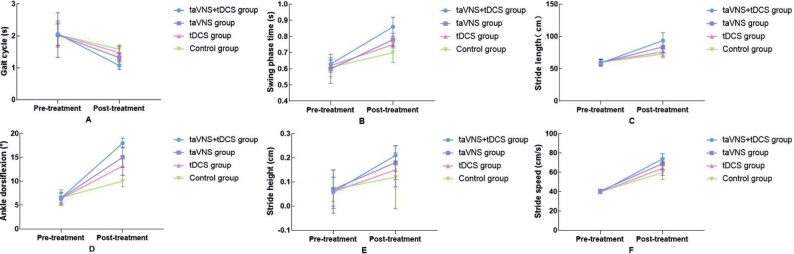
Comparison of gait parameters in 4 groups before and after treatment. (A) Showcases the comparison of gait cycles before and after treatment across 4 groups. (B) Describes changes in swing phase duration. (C) Compares stride length differences. (D) Records adjustments in ankle dorsiflexion. (E) Elucidates variations in stride height. (F) Details the comparison of stride speed before and after treatment.

The study rigorously examined changes in gait parameters across the 4 groups before and after the treatment period. Baseline comparisons showed no significant differences in gait parameters – gait cycle, swing phase time, stride length, ankle dorsiflexion angle, step height, and walking speed – among the 4 groups *p* > 0.05), providing a consistent baseline for subsequent comparisons. Post-treatment analysis revealed noteworthy improvements in all examined gait parameters across the combined, taVNS, and tDCS groups compared with the control group (*p* < 0.05). Most notably, the combined group exhibited significant enhancements in specific parameters: the gait cycle was reduced from 2.06 (0.41) s to 1.07 (0.12) s, swing phase time increased from 0.63 (0.02) s to 0.86 (0.06) s, stride length surged from 58.89 (4.97) cm to 93.57 (12.14) cm, and additional significant improvements included ankle dorsiflexion increasing from 6.52 (0.99°) to 17.98 (1.02°), step height from 0.06 (0.06) m to 0.21 (0.04) m, and walking speed from 39.54 (1.68) cm/s to 73.96 (5.34) cm/s, significantly outperforming the taVNS and tDCS groups individually (*p* < 0.001). These pronounced improvements suggest that the combined application of taVNS and tDCS may offer substantial benefits over single-modality treatments, enhancing gait dynamics more effectively than either therapy alone. The statistical significance of these findings underscores the potential of integrated neuro-modulatory approaches in advancing gait recovery for stroke patients, as detailed in Fig. 4 and Table II.

### Comparative analysis of electromyography before and after treatment among the four groups

**Fig. 5:**
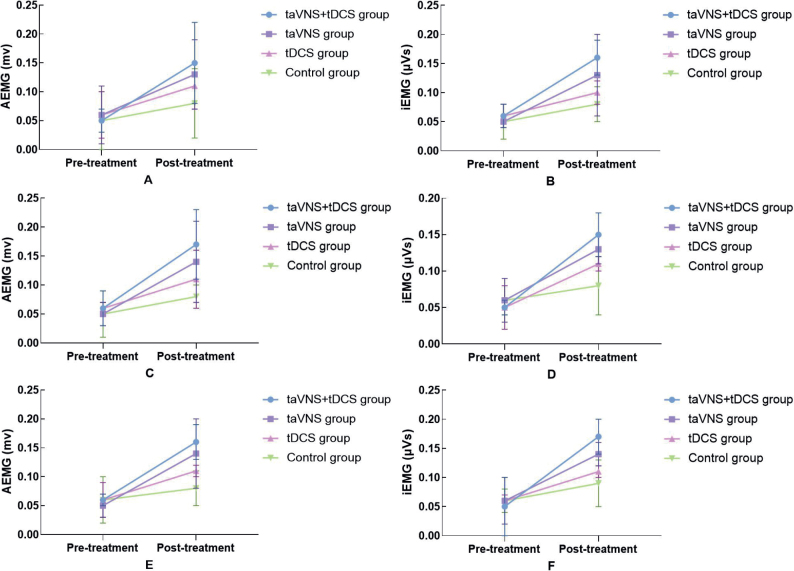
Comparison of EMG in 4 groups before and after treatment. (A, B) Comparison of quadriceps muscle AEMG and iEMG before and after treatment in 4 groups. (C, D) Comparison of anterior tibial muscle AEMG and iEMG before and after treatment in 4 groups. (E, F) Comparison of the lateral head muscle of the gastrocnemius muscle AEMG and iEMG before and after treatment in 4 groups.

The study’s EMG analysis of the quadriceps femoris, tibialis anterior, and lateral head of the gastrocnemius revealed no significant pre-treatment differences among the 4 patient groups, ensuring a reliable baseline for evaluating the interventions’ effectiveness. Post-treatment, the taVNS combined with the tDCS group demonstrated substantial improvements in both AEMG and iEMG values, significantly outperforming the taVNS and tDCS groups. Specifically, AEMG and iEMG values in the combined group increased more markedly – for example, AEMG for the quadriceps femoris rose from 0.05 (0.02) to 0.15 (0.07), tibialis anterior from 0.06 (0.03) to 0.17 (0.06), and lateral gastrocnemius from 0.06 (0.01) to 0.16 (0.03). These results, detailed in Fig. 5 and Table II, underscore the combined treatment’s enhanced impact on muscle activation and coordination, suggesting that integrating taVNS with tDCS may offer significant advantages in muscular function enhancement post-stroke, surpassing the effects of individual treatments or standard care.

## DISCUSSION

This study aimed to evaluate the efficacy of combined taVNS and tDCS in improving gait and lower limb function among subacute stroke patients. After a 4-week treatment period, the study observed significant improvements in the taVNS group, the tDCS group, and the combined taVNS combined with tDCS group across various metrics, as compared with the control group. These metrics included the FMA-LL, MBI, BBS, TUG, and several gait parameters: gait cycle, swing phase time, stride length, ankle dorsiflexion angle, step height, and step speed. Additionally, notable enhancements were seen in the AEMG and iEMG values for the quadriceps femoris, tibialis anterior, and peroneus longus lateral-head muscles. Our findings demonstrate that the combination of taVNS and tDCS resulted in significantly greater improvements in gait parameters, muscle activation, and overall motor function compared with either modality alone or the control group. These results suggest that the synergistic effects of taVNS and tDCS could offer a promising therapeutic approach for enhancing motor recovery in stroke rehabilitation.

Hemiparesis, a common and debilitating consequence of stroke, often leads to altered gait mechanics, such as prolonged swing phase and reduced stance phase on the affected side (23), impairing mobility and increasing the risk of falls, which significantly impacts the quality of life for stroke survivors (1). Despite the potential of traditional rehabilitation techniques, many patients continue to experience persistent gait impairments (24). Neuromodulation techniques like tDCS and taVNS have emerged as promising interventions to enhance the rehabilitation process by promoting neural plasticity and improving motor outcomes. tDCS, known for its safety, simplicity, and tolerability, has been shown to improve gait and balance by enhancing ankle joint movement and restoring symmetry between cerebral hemispheres (25–29). tDCS can restore this symmetry by either stimulating the affected cerebral cortex or inhibiting the healthy side (30, 31), thus enhancing walking ability. Additionally, research indicates that combining taVNS with tDCS can produce a synergistic effect on brain regions, making taVNS a valuable preconditioning tool for enhancing the efficacy of tDCS (17). While tDCS is recommended for treating lower limb dysfunction in stroke patients, taVNS, a non-invasive intervention increasingly favoured by researchers, has primarily been studied for upper limb dysfunction due to its broad activation of the cerebral cortex. However, preliminary studies suggest that taVNS can also improve balance and walking ability in patients with lower limb dysfunction, particularly when used in a closed-loop system, which allows for more effective and efficient treatment by adjusting stimulation parameters in response to physiological changes and patient feedback (32).

Our results revealed that the combined taVNS and tDCS intervention had the most pronounced impact on the EMG values of the quadriceps femoris, tibialis anterior, and lateral head of the peroneus longus muscles of the affected side, followed by the taVNS group. These findings suggest that the synergistic application of taVNS and tDCS could significantly enhance lower limb walking function in stroke patients, more so than either intervention alone. This conclusion underscores the potential of combined neuromodulatory approaches in optimizing post-stroke rehabilitation outcomes. Although recent research on taVNS in lower limb motor function and gait treatment is limited, our study’s gait analysis and electromyography findings indicate that taVNS can significantly improve these aspects in post-stroke patients even more effectively than tDCS. Previous research has demonstrated that taVNS can activate brainstem nuclei and modulate cortical networks that are crucial for motor control and neuroplasticity (15). Similarly, tDCS has been shown to enhance motor function by increasing corticospinal excitability through the depolarization of cortical neurons in the M1 area (33, 34). However, research specifically addressing the combined effects of taVNS and tDCS on motor function remains limited. Based on these findings, we hypothesized that combining these 2 modalities would leverage their complementary mechanisms to achieve superior improvements in motor function. This hypothesis was substantiated by our results, which revealed significant enhancements in gait parameters, including stride length, walking speed, and muscle activation, as comprehensively assessed by sEMG, gait analysis, and motor function evaluation.

In this study, we employed a wearable gait analysis system and sEMG for the comprehensive evaluation of gait in stroke patients. The wearable gait analysis system, recognized for its precision and objectivity, is an innovative tool in gait research. It facilitates the analysis of both spatial and temporal gait parameters such as step speed, frequency, length, amplitude, and width, as well as gait cycle and timing. Additionally, it provides insights into biomechanical parameters, including joint angles at the hip, knee, and ankle of the affected limb (35). These parameters, particularly step speed and length, are crucial in assessing the walking capabilities of post-stroke patients. Numerous studies have underscored the role of enhanced joint mobility in the affected hip, knee, and ankle in improving walking ability through rehabilitation therapy (36). In our study, measurements of step speed, stride length, step height, and ankle dorsiflexion angle further validated their association with gait improvement and walking ability, reinforcing their importance in evaluating walking performance. sEMG, on the other hand, measures the electrical potential generated by muscle fibres during contraction, as detected by electrodes placed over the skin. This method reflects the collective activity of multiple motor units over time and space, offering insights into the correlation between muscle activity and functional state (37). As a non-invasive, objective, and quantitative assessment tool, sEMG is extensively utilized to evaluate motor dysfunction severity in stroke patients.

Our study observed no significant clinical changes in cardiovascular parameters during the treatment process, and no adverse reactions were reported. Therefore, we conclude that taVNS and tDCS are safe, well-tolerated treatments with rare adverse events, offering promising avenues for the treatment of various conditions influenced by vagus nerve activity.

### Limitations

The study, while providing valuable insights, does have certain limitations that need acknowledgment and consideration.

*Limited sample size and scope.* The study’s sample size was relatively small and confined to subacute stroke patients. Consequently, the findings may not be generalizable to the broader stroke population. Stroke recovery, particularly in its acute and subacute phases, is characterized by a high degree of variability and complexity. The diverse nature of stroke presentations and the potential for spontaneous recovery in these stages necessitate further research with a larger and more varied patient cohort to validate and extend these findings.

*Optimization of stimulation parameters.* One of the most significant challenges in this study pertains to determining the optimal stimulation parameters for both taVNS and tDCS. These parameters, including intensity, duration, and location of stimulation, profoundly influence clinical outcomes. Given that taVNS and tDCS are relatively new in the realm of non-invasive neural stimulation techniques, their application in clinical settings is still evolving. The underlying principles and mechanisms are not fully understood, and there is currently no standardized protocol for setting these stimulation parameters. Further research is required to establish more definitive guidelines that could enhance the efficacy and applicability of these techniques in clinical practice.

### Conclusion

These results collectively suggest that taVNS, tDCS, and their combination can effectively enhance gait, walking ability, and overall quality of daily life in stroke patients. Notably, the combined treatment of taVNS and tDCS, a relatively understudied approach, demonstrated promising results as a rehabilitation therapy for improving the gait of stroke patients. Furthermore, the absence of reported adverse events during the treatment process underscores the safety and tolerability of these interventions. The findings of this study contribute valuable insights into stroke rehabilitation, particularly highlighting the potential benefits of combining taVNS and tDCS as a therapeutic strategy to address gait impairments in stroke patients.
